# Exploring 1400 Plasma Metabolites and Polycystic Ovary Syndrome: A Bidirectional Mendelian Randomization Study

**DOI:** 10.1002/brb3.70745

**Published:** 2025-08-04

**Authors:** Xiaoli Lei, Xiaobei Liu, Yi Qu, Yanqin Huang

**Affiliations:** ^1^ The First Clinical Medical College Shandong University of Traditional Chinese Medicine Jinan China; ^2^ Department of Reproductive Genetics Taian Central Hospital Taian China; ^3^ Department of Endocrinology Affiliated Hospital of Shandong University of Traditional Chinese Medicine Jinan China

**Keywords:** 1400 plasma metabolites, causality, Mendelian randomization, polycystic ovary syndrome

## Abstract

**Background:**

Evidence concerning the causal effects of blood metabolites on polycystic ovary syndrome (PCOS) risk remains scarce. A two‐sample Mendelian randomization (MR) analysis was performed among individuals of European ancestry to establish a causal relationship between plasma metabolites and the risk of PCOS.

**Objective:**

To determine causal associations between 1400 blood metabolites and PCOS.

**Methods:**

Utilizing data from a GWAS, which encompasses over 1400 blood metabolites, bidirectional MR was employed to explore the potential causal associations between PCOS and these metabolites. Causal inference methods included inverse variance weighting, MR‐Egger regression, weighted median, weighted mode, and simple mode approaches. Sensitivity analyses were performed to validate the robustness of the results. Reverse MR analyzed 1400 blood metabolites as outcomes and PCOS as the exposure.

**Results:**

Fifteen plasma metabolites, including methionine sulfoxide, N‐acetylserine, and glycine, exhibited positive causal effects on PCOS risk. In contrast, 13 metabolites, including threonate, 7‐methylguanine, and theophylline, demonstrated protective effects. Leave‐one‐out analysis confirmed the stability of the results, with no influential instrumental variables, and eliminated the impacts of heterogeneity and horizontal pleiotropy. Reverse MR revealed no evidence of causal associations between PCOS and the metabolites.

**Conclusion:**

Thirteen plasma metabolites were identified as protective factors for PCOS. Targeted modulation of these metabolites may improve metabolic dysfunction in patients, alleviating symptoms and enhancing quality of life. Further exploration of metabolite dynamics could advance mechanistic insights into PCOS pathogenesis and inform therapeutic development.

## Graphical abstract

Bidirectional Mendelian randomization found 15 plasma metabolites that increase PCOS risk, such as methionine sulfoxide, N‐acetylserine, and glycine, and 13 that offer protection, including threonate, 7‐methylguanine, and theophylline. These results suggest new therapeutic targets for addressing metabolic dysfunction in PCOS.

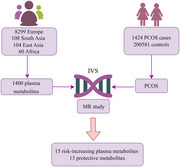

**Abbreviations**
CI: confidence intervals
FSH: follicle‐stimulating hormone
GC: granulosa cell
HA: hyperandrogenism
IR: insulin resistance
IVs: instrumental variables
IVW‐FE: inverse variance weighted‐fixed effects
LH: luteinizing hormone
MR: Mendelian randomization
OR: odds ratios
PARP‐1: poly(ADP‐ribose) polymerase‐1
PCOS: polycystic ovary syndrome
SM: simple mode
SNPs: single‐nucleotide polymorphisms
WM: weighted mode
WME: weighted median method

## 1 Introduction

### 1.1 Background on Polycystic Ovary Syndrome (PCOS)

PCOS is a heterogeneous endocrine disorder with a global incidence ranging from 8% to 13% among women of reproductive age (Zeng et al. 2020; Calcaterra et al. 2021). Among black women, the incidence rate is 8%, whereas among white women, it is 4.8% (Tan and Huang 2019). PCOS is characterized by clinical features such as irregular menstruation, anovulation, acne, hirsutism, and obesity. Additional manifestations include elevated ratios of luteinizing hormone/follicle‐stimulating hormone (LH/FSH), insulin resistance (IR), hyperandrogenism (HA), and enlarged ovaries (Han et al. 2023). The effects of PCOS are chronic, diverse, and lifelong (Ganie et al. 2019). Early in its course, PCOS often presents with infertility and adverse pregnancy outcomes. Long‐term risks include increased rates of endometrial cancer, Type 2 diabetes, and cardiovascular diseases, which significantly impact women's physical and mental health. Follicular fluid analyses reveal PCOS‐specific metabolic disturbances, including elevated oxidative stress markers that impair oocyte maturation (Moreira et al. 2023; Vale‐Fernandes et al. 2025). However, the specific causes and pathophysiology of PCOS remain incompletely understood (Jakubowska‐Kowal et al. 2024; Zhou and Hultgren 2020). There is currently no effective, curative treatment strategy for PCOS; mainly, symptomatic treatments including lifestyle interventions, maturation cycle regulation, testosterone reduction, and metabolism improvement, are employed. About half‐cycle progesterone or short‐acting oral contraceptive combination therapies are used primarily for PCOS patients with ovulatory disorders. However, it cannot reverse spontaneous ovulation in patients with PCOS, and relapse easily occurs after drug withdrawal. Metformin improves metabolic disorders associated with PCOS but has low‐ovulation rates and can cause several adverse reactions, such as diarrhea, nausea, fatigue, and headache (Wishart 2016). Surgical fertility treatments can be considered for infertility; however, while they offer temporary fertility benefits, they do not address the underlying metabolic dysregulation. This therapeutic impasse highlights the urgent need for mechanism‐based interventions targeting the metabolic pathways involved in PCOS pathogenesis.

### 1.2 Significance of Plasma Metabolites in Disease Pathogenesis

Plasma metabolites play crucial roles in influencing disease risk, acting as potential targets for therapeutic interventions (Lejman‐Larysz et al. 2023). Exploring the causal involvement of plasma metabolites in disease pathogenesis offers actionable points for treatment. Observational studies have revealed a notable association between specific blood metabolites and the development of PCOS. Women with PCOS frequently exhibit disruptions in amino acid, carbohydrate, steroid hormone, lipid, and purine metabolism. Abnormal changes in the levels of several blood metabolites, including vitamin D, have been observed in individuals with PCOS (Yu et al. 2021). Z. Zhang et al. (2020) identified disruptions in sphingolipid metabolism and phenylalanine, tyrosine, and tryptophan biosynthesis, along with glutathione metabolism, in females diagnosed with PCOS (Chang et al. 2017). Srnovršnik et al. (2023) observed increased levels of branched‐chain amino acids (valine, isoleucine, and leucine) in obese females with PCOS. Lazúrová et al. (2021) found that blood levels of BPA were significantly higher in women with PCOS, positively correlated with androgens but negatively correlated with ovarian steroid hormones. Milankov et al. (2023) reported that higher concentrations of phthalate metabolites could disrupt obesity, glucose, and lipid metabolism in women with PCOS. Establishing links between plasma metabolites and human diseases is crucial for advancing prevention, diagnosis, and treatment strategies related to these metabolites.

### 1.3 Rationale for the Mendelian Randomization (MR) Approach

MR functions similarly to a randomized controlled trial, using single‐nucleotide polymorphisms (SNPs) as instrumental variables (IVs) to infer causal associations with outcomes. Observational epidemiological studies are prone to bias, confounding factors, and reverse causality (Evans and Davey Smith 2015). However, MR analysis can mitigate bias from confounding factors and reverse causal associations (Gupta et al. 2017; Lawlor et al. 2008). In this study, bidirectional MR methodology was employed to investigate the causal associations between plasma metabolites and polycystic ovaries.

### 1.4 Study Hypothesis

This study included samples from both European and non‐European populations, thereby improving the generalizability and comprehensiveness of the findings. The total number of PCOS cases was 1424, with 200,581 controls, which represents a significantly larger sample size than those reported in previous MR studies of similar scope and helps reduce the potential for sampling bias. The use of bidirectional MR methodology allows for a more comprehensive evaluation of causal relationships by examining both forward and reverse causality, thus overcoming the limitations associated with traditional unidirectional analyses. We propose the following hypothesis: plasma metabolites, including amino acids, carbohydrates, steroid hormones, lipids, and purine metabolism disruptors, play a causal role in the pathogenesis of PCOS. Through MR analysis, we hypothesize that a causal relationship exists between these plasma metabolites and the development of PCOS. This could facilitate the identification of novel therapeutic targets and lead to more effective treatment strategies for patients with PCOS.

## 2 Materials and Methods

### 2.1 Design

**FIGURE 1:**
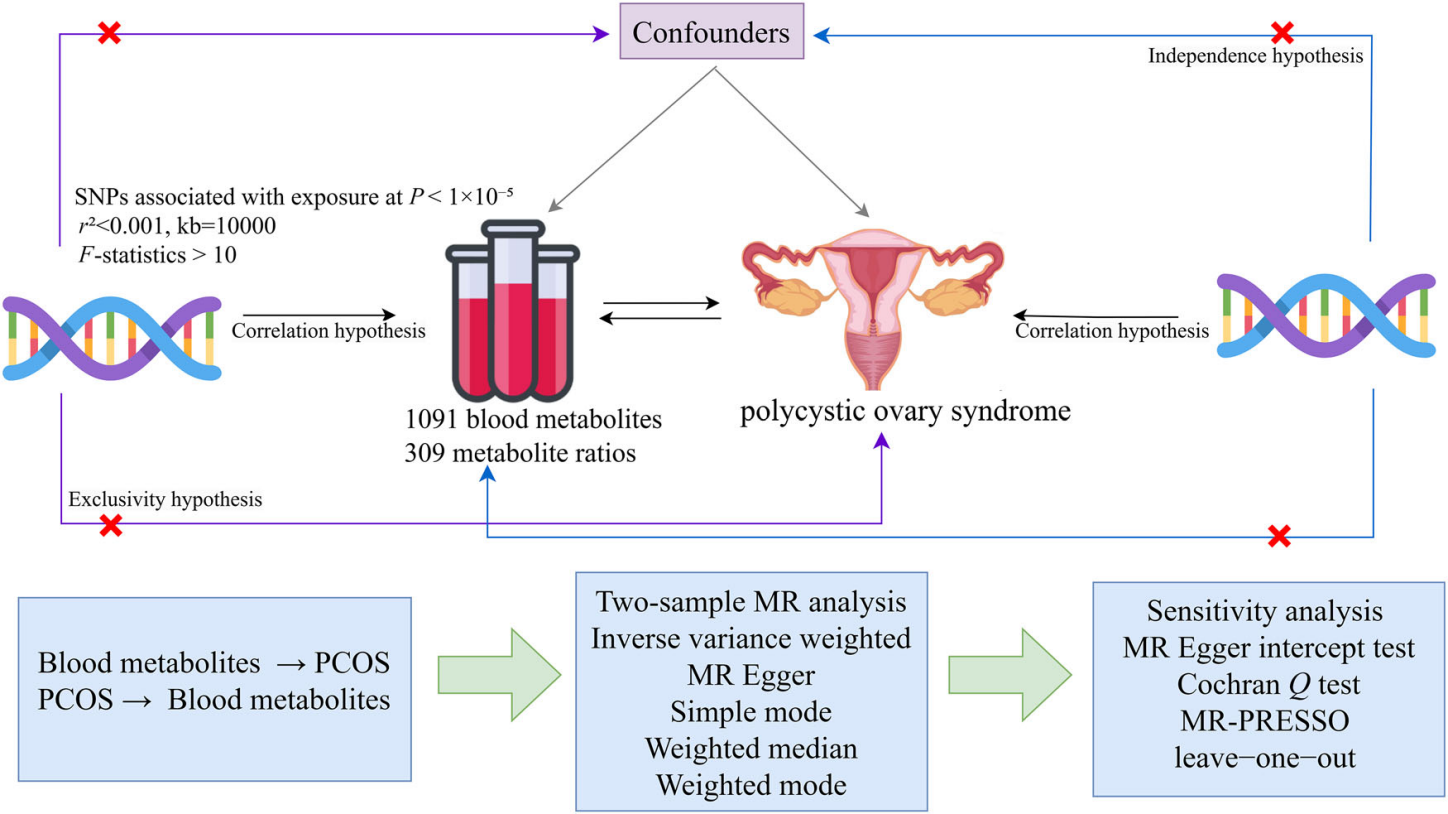
Illustration of the comprehensive framework of MR research.

The present study employed two‐sample MR analysis to investigate the causal link between plasma metabolites and PCOS. Genome‐wide association data encompassing 1400 plasma metabolites and PCOS patients were gathered. To ensure robustness, three critical assumptions were upheld for MR: (1) IV SNPs used in the analysis were highly associated with exposures (1400 plasma metabolites or PCOS); (2) SNPs were independent of known confounders affecting 1400 plasma metabolites or PCOS; and (3) SNPs influenced the outcome of PCOS or 1400 plasma metabolites solely through exposure to either 1400 plasma metabolites or PCOS. Please refer to Figure 1.

### 2.2 Data Sources

A total of 1400 plasma metabolites were identified across four cohorts, encompassing 8299 individuals in Europe, 108 in South Asia, 104 in East Asia, and 60 in Africa, with access to approximately 15.4 million SNPs through GWAS (https://www.ebi.ac.uk/gwas/). The European GWAS is accessible under accession numbers GCST90199621‐90201020, whereas non‐European GWAS data can be found under CST90201021‐90204063 (Chen et al. 2023). In addition, the Finngen database (https://finngen.gitbook.io/documentation/) provides data on 1424 PCOS patients and 200,581 control subjects, along with information on 18703040 SNPs specific to PCOS within a European population of European ancestry. All summary data used in this study are publicly available for download. Ethical approval was obtained from the respective institutions for each included GWAS.

### 2.3 Tool Variable Selection

To identify IVs, SNPs showing strong associations with 1400 plasma metabolites at a significance threshold of *p* < 1.0 × 10^−5^ were selected. Redundant SNPs were pruned using linkage disequilibrium analysis based on European genomic data, retaining only independent variants with *r* ^2^ < 0.001 within a 10,000 kilobase window. Instrument strength was evaluated using *F* statistics, excluding SNPs with *F* < 10 to avoid weak instrument bias. Palindromic SNPs with ambiguous strand alignment were removed to prevent allele miscalculation. All candidate SNPs were systematically screened via PhenoScanner (http://www.phenoscanner.medschl.cam.ac.uk/) to assess and eliminate variants with known associations to potential confounding traits.

### 2.4 Analysis Method

#### 2.4.1 MR Analysis

The inverse variance weighted‐fixed effects (IVW‐FE) model served as the primary method for MR analysis. IVW does not involve the intercept term and assigns weights based on the reciprocal of each IV's variance (Burgess et al. 2013). IVs are used to control for confounding factors when exploring causal relationships between variables. When all IVs are valid and pleiotropy is absent, SNP effects are estimated using weighted regression via the ratio method to derive an overall estimate. This approach, IVW, assumes that the SNPs used as IVs affect results exclusively through exposure‐related pathways. In addition, MR‐Egger regression, the weighted median method (WME), the simple mode (SM), and the weighted mode (WM) can complement IVW. MR‐Egger differs in that it considers the intercept term during regression, with weights based on reciprocals of outcome variances for fitting. WME requires more than 50% validity among IVs, ranking SNPs by descending weights and using the median as the result—an approach that provides reliable estimates of consistent causal effects. SM and WM cluster SNPs with similar effects to estimate causal effects accordingly.

#### 2.4.2 Sensitivity Analysis

1. The Cochran *Q* test was used to evaluate the presence of heterogeneity among the IVs. A *p* value greater than 0.05 indicates a reduced likelihood of heterogeneity (Jaitner et al. 2024).
2. The MR‐Egger intercept test was conducted to evaluate the presence of horizontal pleiotropy. The statistical significance of the intercept term indicates that significant horizontal pleiotropy was observed in this study (Bowden et al. 2015).
3. Mendelian randomization (MR)‐PRESSO values were utilized to detect and correct for pleiotropy and to identify and remove outliers if present (Verbanck et al. 2018).
4. A leave‐one‐out sensitivity analysis was conducted by systematically excluding each SNP to assess its impact on the overall causal effect (Guo and Li 2023).

Moreover, a reverse MR analysis was executed to investigate the presence of a causal association in the opposite direction between the 1400 plasma metabolites identified in the initial MR analysis and PCOS.

### 2.5 Statistical Methods

All the statistical analyses were conducted via the “TwoSampleMR” package in R software version 4.3.2. The results are reported as odds ratios (OR) with corresponding 95% confidence intervals (CI). The significance level was set at *α* = 0.05.

## 3 Results

### 3.1 Causal Effect of 400 Plasma Metabolites on PCOS

**FIGURE 2:**
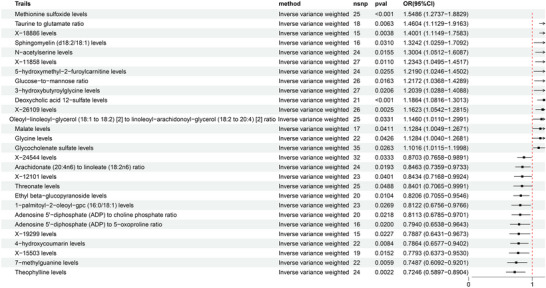
Forest plot of plasma metabolite effect values based on inverse variance weighting and PCOS.

The objective of this study was to establish the causal relationship between 1400 plasma metabolites and PCOS. Following thorough screening for IVs, researchers identified 27,264 SNPs associated with 1294 plasma metabolites. Notably, *F* statistics indicated that one SNP exceeded the threshold of 10, demonstrating robustness against bias from weak IVs. The primary method used was inverse variance weighting, and MR analysis of 1400 plasma metabolites was conducted with a significance threshold of *p* < 0.05, as depicted in Figure 2 and Table 1.

**TABLE 1:** Plasma metabolites causally associated with PCOS based on inverse variance weighting.

Plasma metabolites	nsnp	β	se	or	*p* value
Methionine sulfoxide levels	25	0.4374	0.0997	1.5486	0.0000
Taurine to glutamate ratio	18	0.3787	0.1386	1.4604	0.0063
X‐18886 levels	15	0.3366	0.1162	1.4001	0.0038
Sphingomyelin (d18:2/18:1) levels	16	0.2808	0.1302	1.3242	0.0310
N‐acetylserine levels	24	0.2627	0.1085	1.3004	0.0155
X‐11858 levels	27	0.2105	0.0828	1.2343	0.0110
5‐hydroxymethyl‐2‐furoylcarnitine levels	24	0.1980	0.0886	1.2190	0.0255
Glucose‐to‐mannose ratio	26	0.1965	0.0818	1.2172	0.0163
3‐hydroxybutyroylglycine levels	27	0.1856	0.0802	1.2039	0.0206
Deoxycholic acid 12‐sulfate levels	21	0.1709	0.0472	1.1864	0.0003
X‐26109 levels	26	0.1504	0.0498	1.1623	0.0025
Oleoyl‐linoleoyl‐glycerol (18:1 to 18:2) [2] to linoleoyl‐arachidonoyl‐glycerol (18:2 to 20:4) [2] ratio	25	0.1363	0.0640	1.1460	0.0331
Malate levels	17	0.1208	0.0591	1.1284	0.0411
Glycine levels	22	0.1208	0.0596	1.1284	0.0426
Glycocholenate sulfate levels	35	0.0968	0.0436	1.1016	0.0263
X‐24544 levels	32	−0.1389	0.0653	0.8703	0.0333
Arachidonate (20:4n6) to linoleate (18:2n6) ratio	24	−0.1669	0.0713	0.8463	0.0193
X‐12101 levels	23	−0.1703	0.0830	0.8434	0.0401
Threonate levels	25	−0.1742	0.0884	0.8401	0.0488
Ethyl beta‐glucopyranoside levels	20	−0.1977	0.0772	0.8206	0.0104
1‐palmitoyl‐2‐oleoyl‐gpc (16:0/18:1) levels	23	−0.2080	0.0940	0.8122	0.0269
Adenosine 5′‐diphosphate (ADP) to choline phosphate ratio	20	−0.2092	0.0912	0.8113	0.0218
Adenosine 5′‐diphosphate (ADP) to 5‐oxoproline ratio	16	−0.2307	0.0992	0.7940	0.0200
X‐19299 levels	15	−0.2374	0.1042	0.7887	0.0227
4‐hydroxycoumarin levels	22	−0.2403	0.0911	0.7864	0.0084
X‐15503 levels	19	−0.2493	0.1027	0.7793	0.0152
7‐methylguanine levels	22	−0.2894	0.1052	0.7487	0.0059
Theophylline levels	24	−0.3221	0.1051	0.7246	0.0022

### 3.2 Positive Correlation With PCOS

**FIGURE 3:**
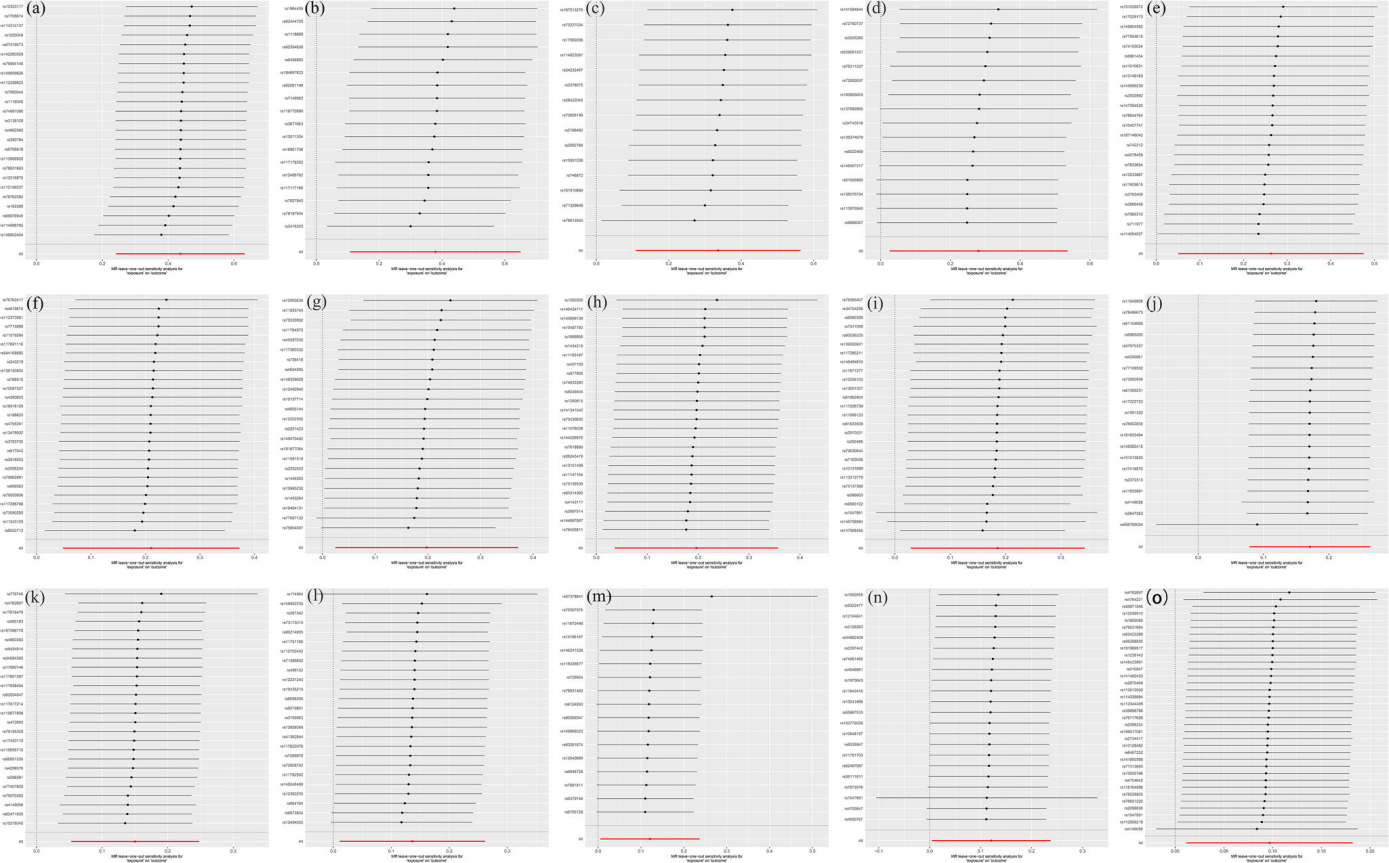
Scatter plot of positive causalities between 1400 plasma metabolites and PCOS. (a) Represents the level of methionine sulfoxide; (b) denotes the taurine‐to‐glutamate ratio; (c) signifies the X‐18886 level; (d) indicates the sphingomyelin (d18:2/18:1) level; (e) corresponds to the N‐acetylserine level; (f) represents the X‐11858 level; (g) denotes the 5‐hydroxymethyl‐2‐furoylcarnitine level; (h) signifies the glucose‐to‐mannose ratio; (i) corresponds to the 3‐hydroxybutyroylglycine level; and (j) represents the deoxycholic acid 12‐sulfate level. In addition, (k) denotes the X‐26109 level, (l) signifies the oleoyl‐linoleoyl‐glycerol (18:1–18:2) [2] to linoleoyl‐arachidonoyl‐glycerol (18:2–20:4) [2] ratio, (m) corresponds to the malate level, (n) denotes the glycine level, and (o) signifies the glycocholate sulfate level.

The IVW MR analysis identified significant positive associations between genetically predicted levels of 15 plasma metabolites/ratios and PCOS risk (*p* < 0.05 for all), with the strongest associations observed for methionine sulfoxide (OR = 1.5486, 95% CI: 1.2737–1.8829, *p* < 0.001), the taurine‐to‐glutamate ratio (OR = 1.4604, 95% CI: 1.1129–1.9163, *p* = 0.0063), and X‐18886 levels (OR = 1.4001, 95% CI: 1.1149–1.7583, *p* = 0.0038); significant positive associations were also found for sphingomyelin (d18:2/18:1) (OR = 1.3242, 95% CI: 1.0259–1.7092, *p* = 0.0310), N‐acetylserine (OR = 1.3004, 95% CI: 1.0512–1.6087, *p* = 0.0155), X‐11858 (OR = 1.2343, 95% CI: 1.0495–1.4517, *p* = 0.0110), 5‐hydroxymethyl‐2‐furoylcarnitine (OR = 1.2190, 95% CI: 1.0246–1.4502, *p* = 0.0255), glucose‐to‐mannose ratio (OR = 1.2172, 95% CI: 1.0368–1.4289, *p* = 0.0163), 3‐hydroxybutyroylglycine (OR = 1.2039, 95% CI: 1.0288–1.4088, *p* = 0.0206), deoxycholic acid 12‐sulfate (OR = 1.1864, 95% CI: 1.0816–1.3013, *p* = 0.0003), X‐26109 (OR = 1.1623, 95% CI: 1.0542–1.2815, *p* = 0.0025), oleoyl‐linoleoyl‐glycerol (18:1–18:2) to linoleoyl‐arachidonoyl‐glycerol (18:2–20:4) ratio (OR = 1.1460, 95% CI: 1.0110–1.2991, *p* = 0.0331), malate (OR = 1.1284, 95% CI: 1.0049–1.2671, *p* = 0.0411), glycine (OR = 1.1284, 95% CI: 1.0040–1.2681, *p* = 0.0426), and glycocholenate sulfate (OR = 1.1016, 95% CI: 1.0115–1.1998, *p* = 0.0263), where an OR > 1 indicates increased PCOS risk per unit increase in the genetically predicted metabolite level or ratio, and *p* < 0.05 denotes statistical significance, as depicted in Figure 3.

**FIGURE 4:**
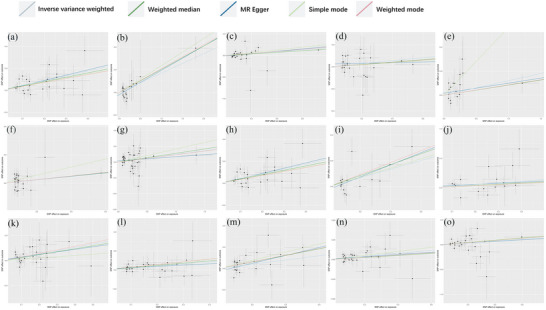
Leave‐one‐out test of the positive causal relationship between 1400 plasma metabolites and PCOS. (a) Represents the level of methionine sulfoxide; (b) denotes the taurine‐to‐glutamate ratio; (c) signifies the X‐18886 level; (d) indicates the sphingomyelin (d18:2/18:1) level; (e) corresponds to the N‐acetylserine level; (f) represents the X‐11858 level; (g) denotes the 5‐hydroxymethyl‐2‐furoylcarnitine level; (h) signifies the glucose‐to‐mannose ratio; (i) corresponds to the 3‐hydroxybutyroylglycine level; and (j) represents the deoxycholic acid 12‐sulfate level. In addition, (k) denotes the X‐26109 level, (l) signifies the oleoyl‐linoleoyl‐glycerol (18:1–18:2) [2] to linoleoyl‐arachidonoyl‐glycerol (18:2–20:4) [2] ratio, (m) corresponds to the malate level, (n) denotes the glycine level, and (o) signifies the glycocholate sulfate level.

Sensitivity analyses confirmed robustness, as Cochran's *Q* test revealed no significant heterogeneity (*p* > 0.05, indicating consistency across IVs), MR‐Egger regression showed no significant horizontal pleiotropy (intercept *p* > 0.05, suggesting confounding pathways do not drive these findings). Leave‐one‐out analysis identified no influential outliers (Figure 4), with detailed results provided in Table 2.

**TABLE 2:** Results from a positive causal sensitivity analysis of 1400 plasma metabolites and their association with PCOS.

Plasma metabolites	heterogeneity		pleiotropy		
	MR Egger Q_pval	IVW Q_pval	MR Egger intercept	MR Egger se	MR Egger intercept pval
Methionine sulfoxide levels	0.6768	0.7267	−0.0059	0.0266	0.8273
Taurine to glutamate ratio	0.2773	0.3375	−0.0036	0.0407	0.9314
X‐18886 levels	0.5839	0.6059	−0.0211	0.0255	0.4235
Sphingomyelin (d18:2/18:1) levels	0.4191	0.4857	0.0194	0.0585	0.7448
N‐acetylserine levels	0.9650	0.9749	0.0085	0.0280	0.7657
X‐11858 levels	0.9857	0.9892	0.0118	0.0255	0.6492
5‐hydroxymethyl‐2‐furoylcarnitine levels	0.2841	0.2849	−0.0262	0.0272	0.3455
Glucose‐to‐mannose ratio	0.8445	0.8715	0.0095	0.0225	0.6749
3‐hydroxybutyroylglycine levels	0.2393	0.2811	0.0063	0.0255	0.8063
Deoxycholic acid 12‐sulfate levels	0.9355	0.9054	−0.0190	0.0148	0.2126
X‐26109 levels	0.4037	0.4339	−0.0105	0.0156	0.5058
Oleoyl‐linoleoyl‐glycerol (18:1–18:2) [2] to linoleoyl‐arachidonoyl‐glycerol (18:2–20:4) [2] ratio	0.4325	0.3648	0.0253	0.0169	0.1486
Malate levels	0.3734	0.3878	0.0165	0.0187	0.3920
Glycine levels	0.6815	0.7376	0.0002	0.0137	0.9876
Glycocholenate sulfate levels	0.7677	0.7637	0.0151	0.0151	0.3233

### 3.3 Negative Correlation With PCOS

**FIGURE 5:**
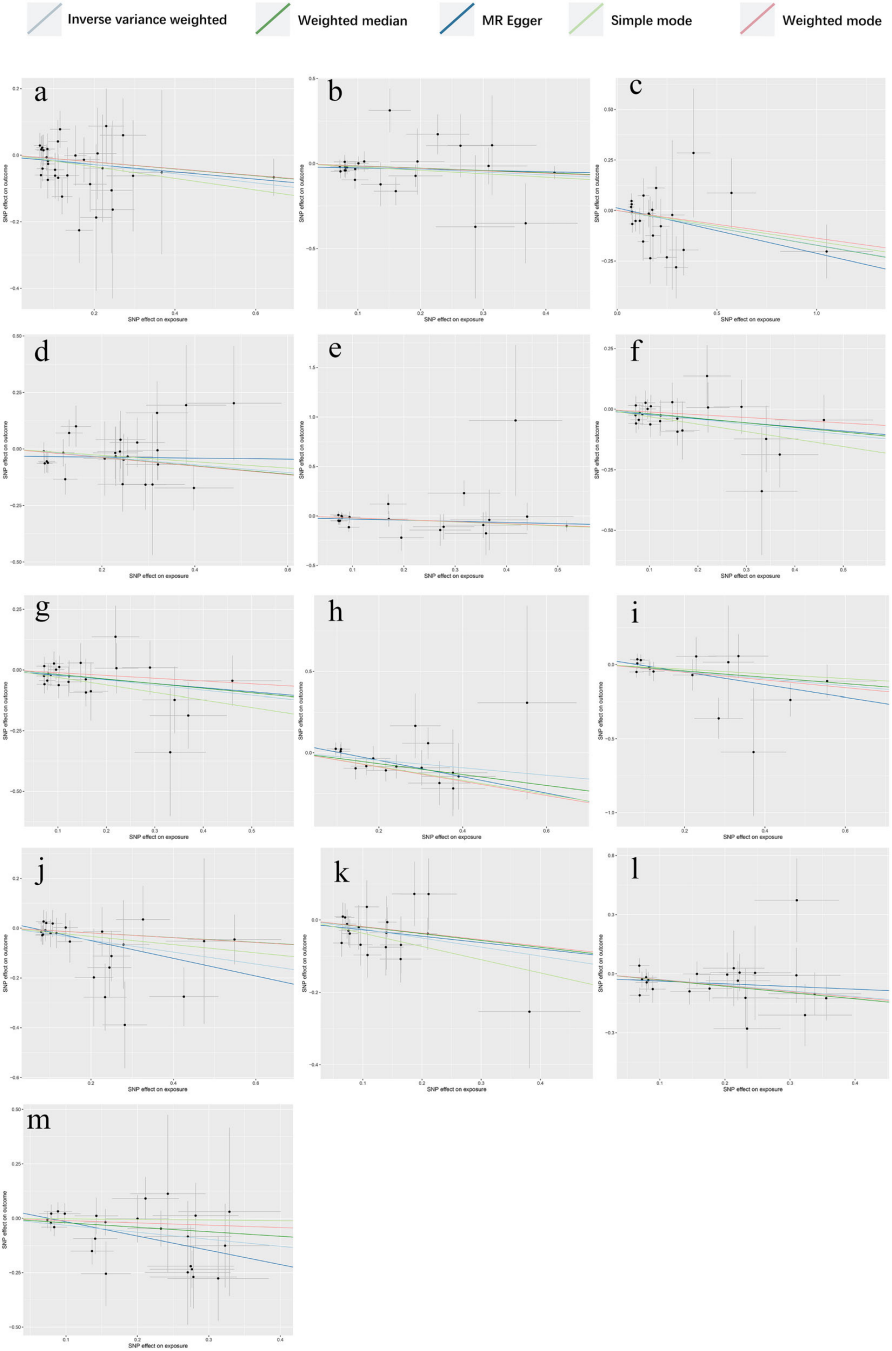
Scatter plot of 1400 plasma metabolites and reverse causality of PCOS. (a) Represents the X‐24544 level; (b) denotes the ratio of arachidonate (20:4n6) to linoleate (18:2n6); (c) signifies the X‐12101 level; (d) corresponds to the threonine level; (e) indicates the ethyl beta‐glucopyranoside level; (f) represents the 1‐palmitoyl‐2‐oleoyl‐gpc (16:0/18:1) level; (g) denotes the adenosine 5′‐diphosphate (ADP) to choline phosphate ratio; (h) signifies the adenosine 5′‐diphosphate (ADP) to 5‐oxoproline ratio; (i) corresponds to the X‐19299 level; (j–m) represent the 4‐hydroxycoumarin, X‐15503,7‐methylguanine, and theophylline levels, respectively.

IVW MR analysis identified significant inverse associations between genetically predicted levels of 13 plasma metabolites/ratios and PCOS risk (*p* < 0.05 for all), indicating protective effects, with the strongest associations observed for theophylline levels (OR = 0.7246, 95% CI: 0.5897–0.8904, *p* = 0.0022), 7‐methylguanine levels (OR = 0.7487, 95% CI: 0.6092–0.9201, *p* = 0.0059), and 4‐hydroxycoumarin levels (OR = 0.7864, 95% CI: 0.6577–0.9402, *p* = 0.0084); additional significant inverse associations were found for X‐15503 levels (OR = 0.7793, 95% CI: 0.6373–0.9530, *p* = 0.0152), X‐19299 levels (OR = 0.7887, 95% CI: 0.6431–0.9673, *p* = 0.0227), adenosine 5'‐diphosphate (ADP) to 5‐oxoproline ratio (OR = 0.7940, 95% CI: 0.6538–0.9643, *p* = 0.0200), ethyl beta‐glucopyranoside levels (OR = 0.8206, 95% CI: 0.7055–0.9546, *p* = 0.0104), 1‐palmitoyl‐2‐oleoyl‐gpc (16:0/18:1) levels (OR = 0.8122, 95% CI: 0.6756–0.9766, *p* = 0.0269), adenosine 5'‐diphosphate (ADP) to choline phosphate ratio (OR = 0.8113, 95% CI: 0.6785–0.9701, *p* = 0.0218), the X‐12101 ratio (OR = 0.8434, 95% CI: 0.7168–0.9924, *p* = 0.0401), the arachidonate (20:4n6) to linoleate (18:2n6) ratio (OR = 0.8463, 95% CI: 0.7359–0.9733, *p* = 0.0193), the X‐24544 ratio (OR = 0.8703, 95% CI: 0.7658–0.9891, *p* = 0.0333), and the threonate ratio (OR = 0.8401, 95% CI: 0.7065–0.9991, *p* = 0.0488), where an OR < 1 indicates decreased PCOS risk per unit increase in the genetically predicted metabolite level or ratio, and *p* < 0.05 denotes statistical significance, referred to in Figure 5.

**FIGURE 6:**
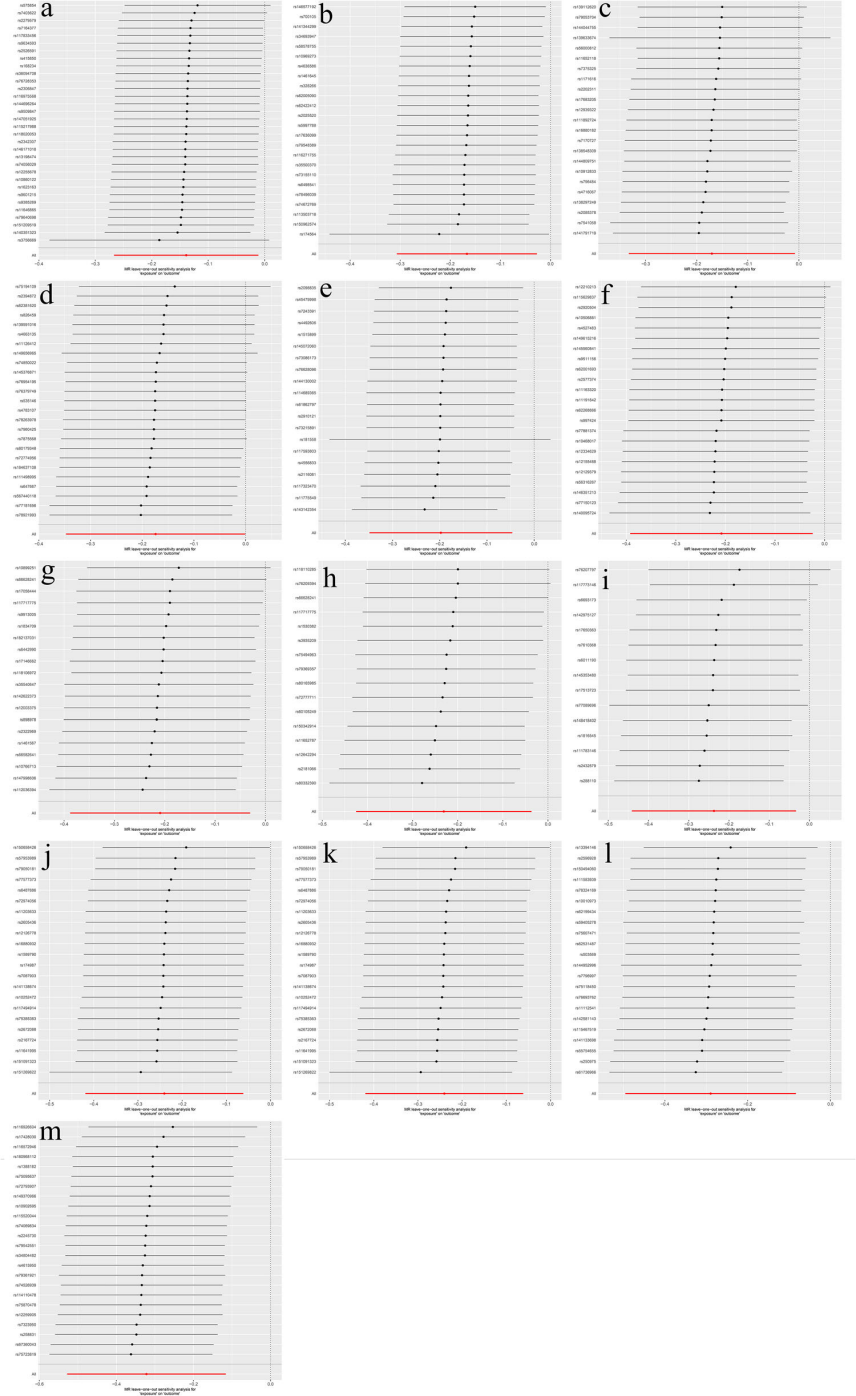
A leave‐one‐out analysis was conducted to investigate the inverse causal relationship between 1400 plasma metabolites and PCOS. (a) Represents the X‐24544 level; (b) denotes the ratio of arachidonate (20:4n6) to linoleate (18:2n6); (c) signifies the X‐12101 level; (d) corresponds to the threonine level; (e) indicates the ethyl beta‐glucopyranoside level; (f) represents the 1‐palmitoyl‐2‐oleoyl‐gpc (16:0/18:1) level; (g) denotes the adenosine 5′‐diphosphate (ADP) to choline phosphate ratio; (h) signifies the adenosine 5′‐diphosphate (ADP) to 5‐oxoproline ratio; (i) corresponds to the X‐19299 level; (j–m) represent the 4‐hydroxycoumarin, X‐15503,7‐methylguanine, and theophylline levels, respectively.

The sensitivity *Q* test indicated no notable heterogeneity among the chosen IVs, whereas MR Egger's analysis confirmed the absence of horizontal pleiotropy for the remaining plasma metabolites (Table 3). Furthermore, the leave‐one‐out method identified no significant outliers, and Figure 6 substantiates the reliability of our MR study findings.

**TABLE 3:** Sensitivity analysis of 1400 plasma metabolites negatively correlated with PCOS.

Plasma metabolites	heterogeneity		pleiotropy		
	MR Egger Q_pval	IVW Q_pval	MR Egger intercept	MR Egger se	MR Egger intercept pval
X‐24544 levels	0.7848	0.8149	−0.0063	0.0150	0.6765
Arachidonate (20:4n6) to linoleate (18:2n6) ratio	0.5213	0.5172	−0.0187	0.0182	0.3147
X‐12101 levels	0.3616	0.3970	0.0125	0.0203	0.5449
Threonate levels	0.7718	0.7554	−0.0318	0.0290	0.2843
Ethyl beta‐glucopyranoside levels	0.4659	0.4622	−0.0200	0.0194	0.3146
1‐palmitoyl‐2‐oleoyl‐gpc (16:0/18:1) levels	0.9511	0.9650	−0.0057	0.0230	0.8071
Adenosine 5′‐diphosphate (ADP) to choline phosphate ratio	0.4554	0.5018	0.0221	0.0399	0.5870
Adenosine 5′‐diphosphate (ADP) to 5‐oxoproline ratio	0.8051	0.7411	0.0541	0.0407	0.2048
X‐19299 levels	0.5163	0.4470	0.0353	0.0256	0.1916
4‐hydroxycoumarin levels	0.8370	0.8395	0.0209	0.0235	0.3844
X‐15503 levels	0.8212	0.8595	−0.0094	0.0277	0.7398
7‐methylguanine levels	0.6368	0.6450	−0.0237	0.0263	0.3773
Theophylline levels	0.6757	0.5691	0.0493	0.0301	0.1151

### 3.4 Reverse MR Analysis Results

**FIGURE 7:**
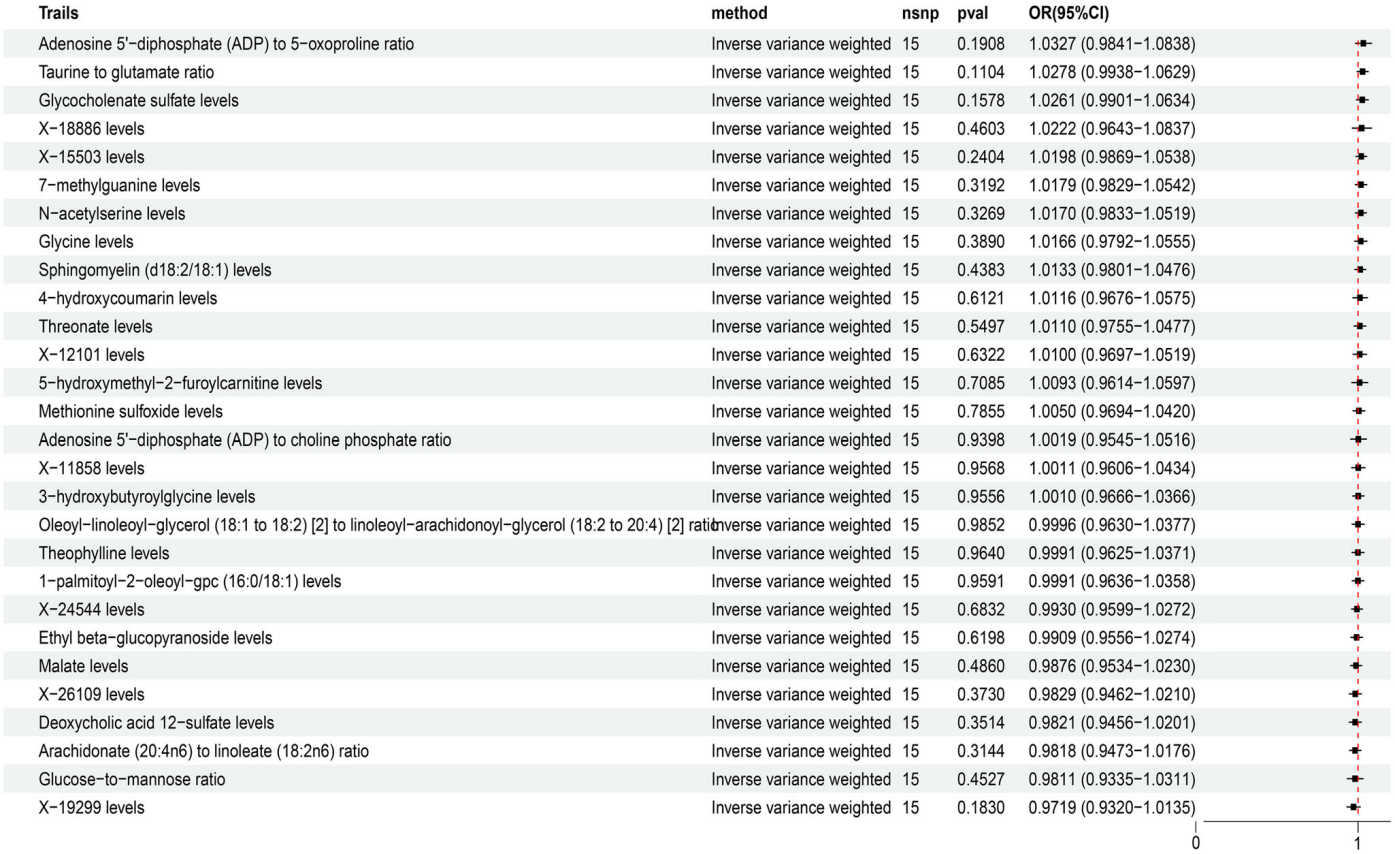
PCOS and 1400 plasma metabolites for reverse MR analysis.

Through the application of reverse MR analysis on a dataset comprising 1400 plasma metabolites and individuals with PCOS, all *p* values obtained from the inverse variance‐weighted method were found to be greater than 0.05, indicating an absence of evidence for a reverse causal relationship, as depicted in Figure 7.

## 4 Discussion

PCOS is linked to metabolic effects such as IR (Behboudi‐Gandevani et al. 2016), dyslipidemia, abnormal glucose metabolism (Anagnostis et al. 2018), and HA (Azziz et al. 2009). Studies have consistently reported abnormal levels of plasma metabolites in PCOS patients, suggesting a close connection with disrupted metabolic function (Özdemir et al. 2010). Understanding the mechanisms linking PCOS and plasma metabolites can provide new perspectives and strategies for managing this condition. Advances in genome‐wide association studies have enhanced measurement accuracy and expanded the coverage of genetic variation, thereby reducing biases from measurement errors in research. This progress provides a robust foundation for investigating how plasma metabolites contribute to the development of PCOS. Genetic variation, which is integral to individual genetic coding and influenced by environmental factors, precedes measurable variables in adults. Causal associations derived from such studies are more reliable than those from observational research and resemble those derived from natural randomized controlled trials. Therefore, this study employed rigorous methodologies to evaluate the causal link between plasma metabolites and PCOS.

The present study utilized a publicly available database containing data on 1400 plasma metabolites and GWAS aggregated statistics on 1400 plasma metabolites associated with PCOS. Two‐sample MR was employed to assess the causal relationship between these plasma metabolites and PCOS. Assuming the validity of all IVs, IVW was used as the primary analytical method to determine causality. The IVW analysis revealed significant positive correlations between methionine sulfoxide concentrations. This study utilized a publicly available database containing data on 1400 plasma metabolites and GWAS aggregated statistics on 1400 plasma metabolites associated with PCOS. Two‐sample MR was employed to evaluate the causal relationship between these plasma metabolites and PCOS. The validity of all IVs and IVW methods was assumed as the primary analytical approach to assess causality. IVW analysis revealed significant positive correlations between methionine sulfoxide (OR = 1.5486, 95% CI: 1.2737–1.8829, *p* < 0.001), N‐acetylserine (OR = 1.3004, 95% CI: 1.0512–1.6087, *p* = 0.0155), glycine levels (OR = 1.1284, 95% CI: 1.0040–1.2681, *p* = 0.0426), and glycocholate sulfate levels (OR = 1.1016, 95% CI: 1.0115–1.1998, *p* = 0.0263) and the occurrence of PCOS.

Furthermore, additional plasma metabolites were positively correlated with PCOS incidence, as determined by IVW analysis. However, X‐24544 (OR = 0.8703, 95% CI: 0.7658–0.9891, *p* = 0.0333), X‐12101 (OR = 0.8434, 95% CI: 0.7168–0.9924, *p* = 0.0401), threonate (OR = 0.8401, 95% CI: 0.7065–0.9991, *p* = 0.0488), 7‐methylguanine (OR = 0.7487, 95% CI: 0.6092–0.9201, *p* = 0.0059), and theophylline (OR = 0.7246, 95% CI: 0.5897–0.8904, *p* = 0.0022) levels were negatively correlated with PCOS. In addition, other plasma metabolites showed negative associations with PCOS incidence based on the IVW analysis results. Sensitivity analysis indicated no significant heterogeneity or multicollinearity among the IVs, suggesting the relative reliability of the results. However, reverse causality analysis did not provide sufficient evidence to support a causal relationship between PCOS and the 1400 plasma metabolites tested. Further validation through additional studies is warranted.

Plasma metabolites may influence PCOS through various pathways, as highlighted by this study. Furthermore, the impact of these metabolites on PCOS can be mediated through multiple biological mechanisms. PCOS is characterized primarily by hormonal dysregulation, including HA, menstrual irregularities, and ovarian morphology marked by multifollicular structures (Joham et al. 2022). Blood represents a valuable source of readily accessible metabolites, which are essential for identifying circulating biomarkers in patients with PCOS. Despite previous research exploring the link between human blood metabolites and PCOS, a comprehensive understanding of their causal relationship requires further extensive and systematic investigation.

Methionine sulfoxidation represents a crucial posttranslational modification of proteins, serving as a regulatory switch for protein activity that influences specific signaling pathways and associated cellular and physiological processes. Increased levels of methionine sulfoxide are observed in response to oxidative stress development (Drazic et al. 2013; Hung et al. 2011; Erickson et al. 2008). Notably, PCOS patients present elevated levels of oxidative stress markers (Rudnicka et al. 2022), with oxidative stress playing a pivotal role in the pathogenesis of PCOS (Papalou et al. 2016; Moreira et al. 2023), which aligns with the findings presented in this study. N‐acetylserine acts as an inducer of cysteine regulation (Verschueren et al. 2024), which is closely linked to homocysteine metabolism. Elevated homocysteine concentrations are associated with PCOS symptoms, highlighting hyperhomocysteinemia as a risk factor for reproductive dysfunction. Furthermore, N‐acetylserine is positively correlated with hypertension (Yan et al. 2022), a condition that is more prevalent among women with PCOS (Joham et al. 2021). These findings provide relevant evidence supporting a potential positive correlation between N‐acetylserine and PCOS. In patients with PCOS, serum malic acid levels increase, whereas succinic acid levels decrease, suggesting that malic acid may influence tricarboxylic acid cycle dynamics, which affects PCOS pathology (Chang et al. 2017). Further support comes from studies showing elevated citrate levels in the follicular fluid of PCOS patients. Citrate, as a central TCA cycle intermediate, reflects associated glycolytic/metabolic disturbances and mitochondrial dysfunction in PCOS (Vale‐Fernandes et al. 2025). The pivotal role of glycine in driving the pathogenesis of PCOS is underscored, suggesting its potential as a molecular biomarker for future diagnostic applications related to this condition (Roychoudhury et al. 2017). Studies have indicated a close association between elevated glycine levels and increased risks of obesity, IR, and metabolic syndrome among individuals with PCOS (Ye et al. 2022).

Threonate facilitates Mg^2+^ influx into cells, increasing the intracellular availability of its conjugated compounds (G. Zhang et al. 2022). Imbalanced Mg^2+^ levels crucially contribute to β‐cell damage and IR by disrupting glucose metabolism, promoting oxidative stress, and triggering inflammatory mediator secretion (Jacobson and Shyng 2020). These findings suggest that threonine may regulate IR through modulating Mg^2+^ levels. However, increasing evidence suggests that IR is central to PCOS pathogenesis and closely associated with long‐term metabolic complications. About 40% of typical PCOS patients develop impaired glucose tolerance or Type 2 diabetes around age 40, which is exacerbated by age and weight gain (Wild et al. 2010). 7‐Methylguanine interacts at the active site of poly(ADP‐ribose) polymerase‐1 (PARP‐1), inhibiting poly ADP‐ribose synthesis, which reorganizes the chromatin structure and recruits DNA repair proteins. By inhibiting the dissociation of PARP‐1 from DNA damage sites within nucleosomes, 7‐methylguanine likely hinders subsequent DNA repair, replication, and transcription (Kirsanov et al. 2022; Nilov et al. 2020). Proliferation, apoptosis induction, and altered functional states induced by 7‐methylguanine influence granulosa cell (GC)‐affected follicle development, dominant follicle selection, ovulation, luteal formation, steroid secretion, and later embryo developmental potential (Colella et al. 2021). By regulating abnormal GC apoptosis processes, 7‐methylguanine may optimize ovulation. Theophylline potentially exerts anti‐inflammatory effects through histone deacetylases activation, where histone deacetylases counteract histone acetyltransferase activity, promoting histone acetylation and gene promoter region transcriptional exposure (Barnes 2010). Chronic low‐grade inflammation can promote PCOS onset and progression, which is correlated with metabolic abnormalities in PCOS patients (Spritzer et al. 2015). These findings suggest that theophylline may mitigate PCOS symptoms through its anti‐inflammatory effects.

In summary, our findings are highly consistent with existing literature on plasma metabolites associated with PCOS, thereby validating the reliability and biological relevance of our results. Further investigation into the specific roles of these metabolites in PCOS pathogenesis may provide valuable insights for the development of innovative diagnostic tools and targeted therapeutic strategies. Certain metabolites, such as methionine sulfoxide, not only show potential as diagnostic biomarkers but also suggest novel therapeutic avenues through their modulation. This finding provides a molecular basis for metabolomics‐driven subtyping of PCOS. Research indicates that these metabolites enable precise stratification of patient subgroups, supporting tailored intervention approaches. Protective metabolites, such as threonate, highlight antioxidant defense and anti‐inflammatory pathways as promising therapeutic targets. The distinct contributions of individual metabolites to disease mechanisms underscore the underlying pathophysiological heterogeneity of PCOS, suggesting that metabolic profiling can guide clinically meaningful personalized interventions. Although genetic epidemiological methods reduce confounding effects, prospective studies are still needed to validate the utility of these metabolic signatures in diagnosis and treatment, particularly regarding how dynamic changes in metabolite levels correspond to clinical phenotypes. Plasma metabolites directly linked to PCOS pathogenesis offer a promising route to improve diagnostic accuracy and develop mechanism‐based therapies, shifting clinical management from symptom‐oriented care toward precision medicine grounded in individual pathophysiology.

This article marks the initial use of MR to examine causal relationships. Unlike individual‐level analysis in experimental and small‐scale studies, this study offers distinct advantages: (1) it leverages genetic data on plasma metabolites and PCOS from the extensive MiBioGen consortium and Finngen database, thereby supporting statistical power for causal associations; and (2) it mitigates potential confounding factors and reverses causality, thereby minimizing resource expenditures. From a genetic standpoint, this study evaluated the potential causal link between plasma metabolites and PCOS, establishing their susceptibility effects.

However, several limitations warrant careful consideration: (1) Although the large‐scale analysis yielded robust statistical associations, the study population was predominantly of European ancestry. Genetic diversity across different demographic groups can influence both the prevalence and clinical manifestations of PCOS. Non‐European populations may possess distinct genetic architectures and gene–environment interactions. For example, metabolic profiles and responses to hormonal fluctuations may differ in Asian or African populations. (2) While the study identifies statistically significant associations, it does not investigate the underlying biological mechanisms. Future research should incorporate both cellular and animal models to elucidate the molecular functions of these metabolites in the pathogenesis of PCOS. (3) Despite offering valuable insights into PCOS diagnosis and treatment, the practical application of these findings is constrained by uncertainty regarding causality. Changes in metabolite levels may represent a consequence rather than a driver of PCOS. Long‐term prospective studies are required to clarify the temporal relationship between metabolic alterations and disease onset. (4) Although the study suggests potential links between PCOS and other metabolic disorders, the precise nature and magnitude of these associations remain to be fully characterized. Unmeasured confounding factors—such as lifestyle patterns, dietary habits, and environmental exposures—may still affect the validity of the results. Future investigations should include comprehensive assessments of lifestyle and environmental influences to better account for these variables and enhance the robustness of the conclusions.

## 5 Conclusion

This study represents the first comprehensive application of MR to investigate the potential causal relationship between PCOS and 1400 plasma metabolites. It identified 15 risk‐associated metabolites, including methionine sulfoxide, N‐acetylserine, glycine, and glycocholate sulfate, with pathogenic implications, as well as 13 protective metabolites, such as threonate, 7‐methylguanine, and theophylline, that demonstrate inhibitory effects against PCOS development. These metabolite signatures offer promising molecular targets for the development of noninvasive diagnostic tools for PCOS. They may serve as novel biomarkers for predicting ovarian dysfunction and associated metabolic complications. Although MR analysis effectively ruled out reverse causation, further investigations are warranted, including functional gain and loss of function experiments at the cellular level, metabolic intervention studies in animal models, and integrative multi‐omics approaches such as metabolic flux analysis and epigenetic regulatory network mapping to elucidate their precise biological mechanisms.

Given the distinct fluctuations observed in these metabolites within the serum of PCOS patients, clinical translation should be prioritized. This includes establishing a metabolomics‐based disease stratification system to support precision medicine and exploring targeted interventions along key metabolic pathways such as personalized nutritional strategies or selective enzyme inhibitors. Future research should focus on deciphering the molecular interactions between these metabolites and critical pathological features of PCOS, particularly sex hormone imbalance and IR. Such mechanistic insights will provide a theoretical foundation for the development of dual‐targeted therapies that can simultaneously correct metabolic dysregulation and improve reproductive outcomes.

## Author Contributions

**Xiaoli Lei**: writing – review and editing, writing – original draft, visualization. **Xiaobei Liu**: methodology, software, validation, supervision. **Yi Qu**: conceptualization, data curation, formal analysis. **Yanqin Huang**: funding acquisition, investigation, project administration, resources.

## Ethics Statement

The ethical considerations are unnecessary as the data is sourced from the “Genomic atlas of the plasma metabolome prioritizes metabolites implicated in human diseases.”

## Conflicts of Interest

The authors declare no conflicts of interest.

## Peer Review

The peer review history for this article is available at https://publons.com/publon/10.1002/brb3.70745.

## Data Availability

The GWAS database (https://www.ebi.ac.uk/gwas/) contains a comprehensive collection of 1400 plasma metabolites. For the European GWAS, the accession number is GCST90199621‐90201020, while for the non‐European GWAS it is CST90201021‐90204063. Relevant data on PCOS can be accessed in the Finngen databases (https://finngen.gitbook.io/documentation/).
